# Roles of lipid droplets and related proteins in metabolic diseases

**DOI:** 10.1186/s12944-024-02212-y

**Published:** 2024-07-19

**Authors:** Zhongyang Zhang, Zhenghang Yu, Dianyuan Liang, Ke Song, Xiangxin Kong, Ming He, Xinxin Liao, Ziyan Huang, Aijia Kang, Rubing Bai, Yixing Ren

**Affiliations:** 1 Department of Gastroenterology, Affiliated Hospital of North Sichuan Medical College, South Maoyuan Road, Shunqing District, Nanchong, Sichuan Province 637000 China; 2 Institute of Hepatobiliary Pancreatic Intestinal Diseases, North Sichuan Medical College, Nanchong, 637000 China; 3 General Surgery, Chengdu XinHua Hospital Affiliated to North Sichuan Medical College, Chengdu, 610000 China

**Keywords:** Lipid droplet, Lipid metabolism, Metabolic diseases

## Abstract

Lipid droplets (LDs), which are active organelles, derive from the monolayer membrane of the endoplasmic reticulum and encapsulate neutral lipids internally. LD-associated proteins like RAB, those in the PLIN family, and those in the CIDE family participate in LD formation and development, and they are active players in various diseases, organelles, and metabolic processes (i.e., obesity, non-alcoholic fatty liver disease, and autophagy). Our synthesis on existing research includes insights from the formation of LDs to their mechanisms of action, to provide an overview needed for advancing research into metabolic diseases and lipid metabolism.

## Background

Lipid droplets (LDs) serve as cellular lipid storage depots. They are pivotal in various physiological processes, encompassing lipid metabolism, cell signaling, as well as energy storage [1]. The past few decades have seen significant progress in LD-related research. LDs do not function as isolated organelles, instead proteins associated with LDs are integral to their function. These proteins participate in the biogenesis, maturation, and catabolism of LDs, as well as autophagy, lipid metabolism, and post-translational modification, implicating them in metabolic diseases such as obesity and diabetes mellitus, and even in cancer [2, 3]. LD-targeted therapeutics are being explored and they offer innovative venues for developing new treatments and improve existing strategies against diseases and for body conditioning.

## Metabolic processes of LDs

LDs possess unique structures dedicated to fat storage in cells. The morphology and abundance of LDs differ among cell types, and their generation processes reflect this variety [4]. LDs mature through lipid synthesis and the action of specific proteins. During hunger or exercise, LDs get degraded, releasing free fatty acids that participate in lipid metabolism [5]. The biogenesis and lipolysis of LDs are stringently regulated under conditions of metabolic balance and health.

### Structure of LDs

LDs are characterized by unique physical structures. They are sometimes referred to as liposomes and oil bodies (adiposomes), particularly in plant studies [6]. LDs range in size from 1 to 100 μm, their hydrophobic core is predominantly composed of neutral lipids, including triglycerides and sterol esters, along with other nonpolar lipids such as diacylglycerol, cholesterol, and monoacylglycerol [7]. A phospholipid monolayer membrane encases these substances. Phosphatidylcholine is the predominant component of the monolayer, followed by other phospholipids like phosphatidylinositol. The monolayer’s components resemble an endoplasmic reticulum (ER) bilayer, with phospholipid polar groups facing outward and acyl chains interacting with the hydrophobic core. The phospholipid monolayer of yeast LDs, determined using a conventional phospholipid analysis through thin-layer chromatography separation and mass spectrometry analysis, contains phosphatidylcholine (57.5%), phosphatidylinositol (21.5%), phosphatidylethanolamine (16.6%), phosphatidylserine (2.1%), phosphatidic acid (1.8%), and lysophospholipids (0.3%). Interestingly, the phosphatidylinositol content was higher and the phosphatidylethanolamine content lower in the LDs compared to their proportions in cell homogenates, indicating that the monolayer LD membrane is not identical to the bilayer membrane of the cell [8]. Over 200 structural and functional proteins, including DGAT2, RAB18, and perilipin (PLIN), are located on LD surfaces, regulating their homeostasis and interactions [9, 10]. For instance, DGAT2 catalyzes triacylglycerol generation, whereas proteins of the perilipin family participate in the metabolism of the RAB18 protein and LDs. [11–15].

Neutral lipids play a crucial role in bioenergetics and membrane biogenesis. Lipotoxicity, resulting from excessive accumulation of fatty acids, glycerolipids, and sterols, underscores the crucial role of neutral lipid synthesis and accumulation within cells [16]. These lipids can induce cellular stress, apoptosis, mitochondrial dysfunction, as well as inflammation. For instance, in the kidney, lipotoxicity causes proximal tubular cell injury and promotes acute kidney injury (AKI) [17]. This process is mediated by the activation of inflammatory pathways, including Toll-like receptors and the NLRP3 inflammasome, as well as the dissipation of mitochondrial membrane potential [18]. Research has shown that inhibiting specific lipid metabolism pathways can reduce lipotoxicity. Once viewed as mere fatty inclusions, LDs are now recognized as fully functional organelles, thanks to research advancements. Lipid droplets are crucial for lipid storage and metabolism, and for protein storage and degradation. Additionally, they participate in various intracellular processes, including membrane transport, and work in close coordination with other organelles [19–22]. Advancements in understanding LD features have enriched knowledge of disease mechanisms in obesity, metabolic liver disease, and neurological disorders [23, 24]. In addition, LDs also have a role in cellular autophagy [25].

**Fig. 1:**
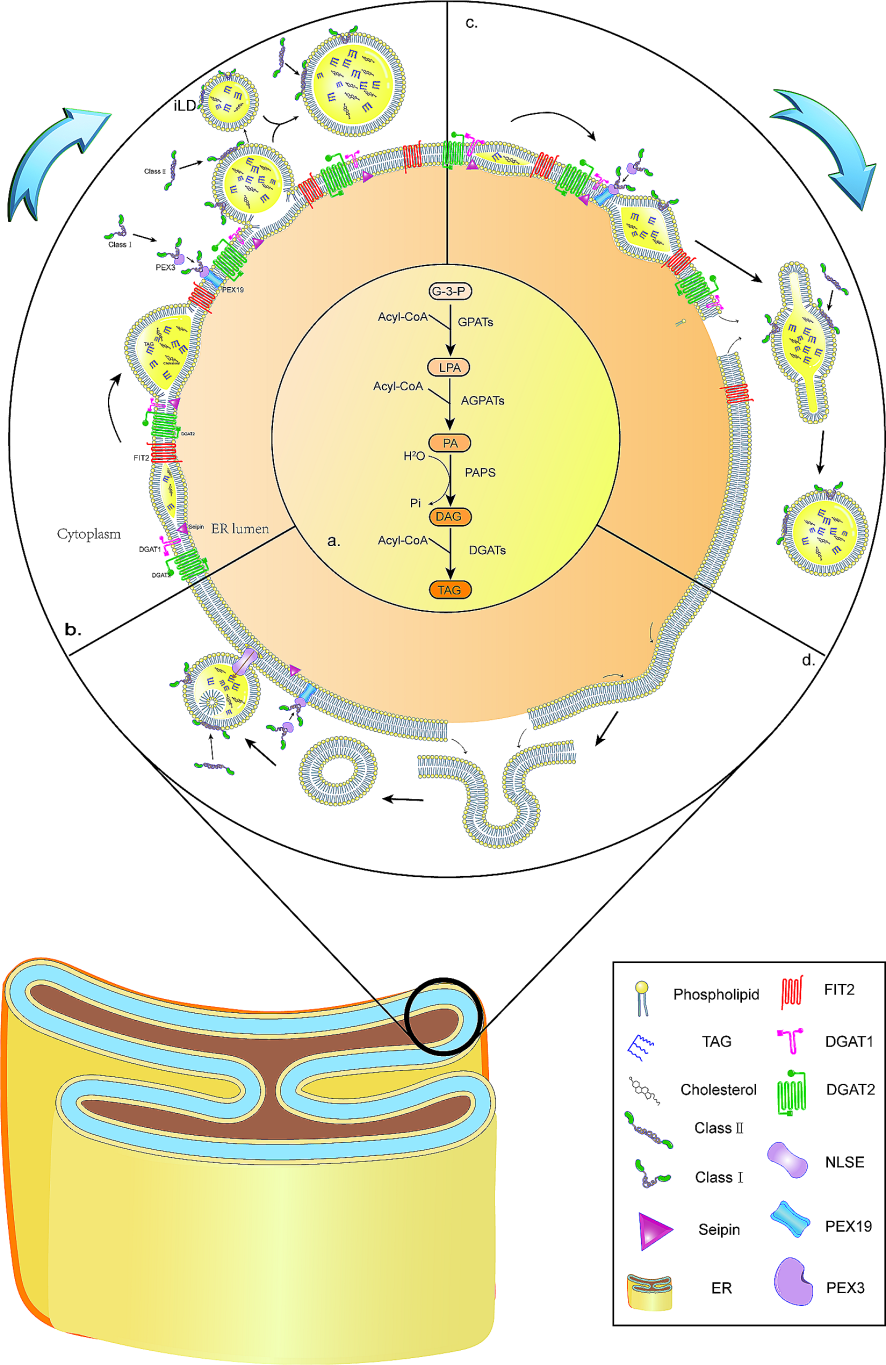
LD biogenesis models. **a**. Schematic representation of triglyceride synthesis within the ER involving key enzymes like GPATs and DGATs, starting from G-3-P (glycerol-3-phosphate). **b**. Budding model of LD formation, where neutral lipid synthesis, nucleation, and budding occur. Following the synthesis of neutral lipids, these lipids accumulate within the ER lipid bilayers, forming a prism-like structure. The synthesis of neutral lipids increases the luminal pressure and results in a monolayered membrane encapsulation that completes the budding. Class I and II proteins associated with the surface of the LDs perform. **c**. Scission model of LD formation. Growing lipid lenses begin to deform the ER membrane which begins to surround the lipids forming structures similar to vesicles that bud off from the ER. Transmembrane proteins are capable of attaching to the membrane by their transmembrane segments. Local curvatures of lipid vesicles and lateral separations between different lipid components occur within the cell. **d**. The vesicular budding model of LDs formation has nascent vesicles near the ER membrane that are connected to the cytoplasmic side. Neutral lipids fill the vesicles either before they are separated from the ER or after budding, through lipid bridges. Subsequently, the residual cavity of each vesicle merges with the external layer of the LDs or remains as an aqueous inclusion within the LDs.

LDs are dynamic organelles within cells that store lipids and regulate their metabolism. Proteins in LDs are classified into classes I and II [26]. Class I proteins, including ACSL3, GPAT4, and FAF2/UBXD8, typically feature a V-shaped hydrophobic hairpin structure. In the absence of LDs, class I proteins are uniformly dispersed within the ER membrane [27]. As an illustration, transporting UBXD8 out of the endoplasmic reticulum towards lipid droplets necessitates involvement by farnesylated peroxisomal biogenesis factor 19 (PEX19) (Fig. 1b) [28]. Class II proteins comprise the perilipin family, choline cytidylyltransferase (CCT), and cell death-inducing DFF45-like effector A (CIDEA). These proteins are synthesized in cytoplasm and attach directly to the surface of LDs (Fig. 1b). For instance, in Drosophila lipid droplets, the phospho-CCT1 targets the surface via its amphipathic helix within the membrane-binding domain. This targeting contributes to the regulation of LD homeostasis, promoting droplet enlargement [29]. The typical structures of the two types of proteins are amphipathic α-helices [30] connected to LDs [31, 32]. These amphipathic α-helices are not exclusive to these proteins, the structures show significant diversity, and the mechanisms guiding them towards LDs remain unclear.

In addition to cytosolic LDs, nuclear and perilipin-coated LDs also form part of the LD family. Studies have recognized nuclear LDs as fully functional organelles, similar in structure to cytosolic ones but differing in their formation mechanism [33]. Nuclear LDs, in contrast to cytosolic ones, have higher levels of cholesterol esters, cholesterol, and phospholipids, lower triglyceride content, and a greater protein-to-lipid ratio [34]. Consequently, nuclear LDs are smaller in volume, but they possess a larger surface area-to-volume ratio compared to regular lipid droplets. Nuclear LDs are linked to heterochromatin, but not necessarily to the nuclear membrane [35].

### Generation of LDs

LDs exhibit similar morphological features throughout a diverse array of species, ranging from bacteria to humans, suggesting a conserved formation process [36]. However, their size, quantity, and distribution vary significantly based on cellular metabolism and availability of nutrients. Enzymes have an essential role in the development and breakdown of LDs, but the specifics of their formation remain elusive. In prokaryotes, LDs originate from the plasma membrane [37], whereas in eukaryotes, they are closely associated with the ER, which occasionally forms a connected or chalazion-like structure around LDs [38]. Several models for LD formation exist, but the budding mechanism is the most widely accepted. Neutral lipid synthesis of triglycerides and sterol esters involves pathways such as the monoglyceride and phosphatidic acid pathways, with enzymes like MGAT1-3, DGAT1,2, and ACA1,2 participating in LD formation [39] (Fig. 1a). After their synthesis, neutral lipids accumulate between the ER’s lipid bilayers. Once the concentration surpasses a critical threshold of approximately 5–10 mol%, they spontaneously segregate and condense into prism-like structures [1, 40]. The budding process in yeast is influenced by proteins such as FIT/FITM, PLIN, seipin, and PEX30 [41, 42]. Seipin is a highly conserved ER membrane protein, and patients with congenital lipodystrophy present seipin mutations [43]. Seipin localization at the nuclear membrane induces accumulation of LDs, suggesting its function in nuclear LDs formation [44]. In addition, seipin participates in phosphatidic acid metabolism, the control of LD size, and the regulation of protein and lipid transport from the ER to LDs. [45]. Two types of FIT proteins exist in mammals: FIT1 and FIT2. FIT2 is similar to lipid phosphatases and phosphotransferases, and it is likely to participate in lipid metabolism [46]. FIT2 depletion leads to accumulation of LDs that are partially revealed to the ER lumen and unable to protrude into the cytosol [47]. This suggests FIT2’s involvement in directing LD budding. Physical factors (like curvatures) induce mechanical stress on accumulating neutral lipids. Curvatures increase triglycerides’ chemical potential and exert a pulling effect on phospholipids, causing packing irregularities or reducing the bilayer interfacial tension. Increases in contact between aqueous molecules and the lipid bilayer’s hydrophobic core act as a physical catalyst for LD assembly [48]. Negative curvature phospholipids like diacylglycerol and phosphatidylethanolamine form embedded structures that inhibit budding, whereas other molecules like lysophospholipids (hemolytic phospholipid) facilitate it (Fig. 1b), these factors influence the development of LDs [49].

The second model describes the removal of LDs from the ER membrane (Fig. 1c). Proteins such as calnexin and immunoglobulin heavy chain-binding protein (BIP) are housed in the ER. Luminal and cytoplasmic leaflets of the ER membrane merge, creating a transient bicellular structure where LDs are encased in a phospholipid monolayer, partially derived from the ER with its associated proteins [50]. Membrane proteins can be accommodated within these structures without completely losing their bilayer properties: LDs form surface ridges featuring localized bilayer membrane structures. These structures anchor to the membrane via their transmembrane segments. Misfolded secreted proteins, marked for degradation, get attached to chaperone proteins like calnexin or BIP. Within cells, lipid vesicles exhibit local bending and lateral separation of lipid components. These visible structures, particularly clear in model membranes used to study LD formation, suggest LDs have defined domains within their structure. The lipid composition and shape of the membrane of LDs may facilitate formation of microdroplet intermediates, or bilayered bisomes, between the luminal and cytoplasmic phospholipid leaflets that give rise to specific lipid interactions and membrane dynamics to form new LDs [51]. However, this model presents an unresolved issue: the generation of LDs leads to instantaneous pore formation and a potential calcium ion imbalance. Additionally, a mechanism for directing LD release exclusively to the cytoplasm, rather than to the ER lumen, remains unclear.

The third model involves vesicle budding, where droplets first form within small bilayer vesicles, leveraging the secretory pathway’s vesicles as the core mechanism (Fig. 1d). These vesicles are specifically used for lipid synthesis. Each new vesicle forms near the ER membrane, connected to its cytoplasmic side. Neutral lipids can traverse the membrane before the vesicle detaches from the ER (post-budding). Lipid bridges populate the vesicles. Subsequently, each vesicle’s residual lumen either merges with the LD’s outer layer or persists within the vesicle as a water inclusion. This model explains the presence of hydrophilic proteins in the core of LDs [52]. Investigations into caveolin-1’s role in LD vesicle formation reveal that it resides within the ER (not outside of it as previously believed) and it integrates into the lipid core during droplet formation. This accounts for the absence of caveolin-1 from the cytoplasm [53].

The traditional budding model is widely accepted and explains the primary process of LD formation from the ER, encompassing initial nucleation, growth, and budding. Its core mechanism is relatively simple, facilitating understanding and experimental verification. However, the precise LD budding mechanisms, in particular the directional budding, remain unclear. The second model offers an in-depth analysis of membrane curvature (shape) and protein roles in LD formation, particularly based on membrane dynamics. This model explains the spatial specificity of the membrane curvature’s impact on LD formation. However, it does not account for instantaneous small-pore calcium imbalances and relies heavily on high-resolution microscopy to observe membrane shapes, making experimental verification challenging. The vesicle budding model innovatively combines vesicle and LD formation, explaining why electron microscopy detects hydrophilic core proteins within LDs. This model is complex, involving multiple protein and molecular interactions, increasing research difficulty.

### Maturation of LDs

Post-budding, LDs interact with the ER, forming membrane bridges that allow triacylglycerol transfer into the LDs [54]. In addition, LDs can directly synthesize triglycerides on their surfaces. As a result of these combined effects, LDs grow and mature [55]. LDs larger than 1–2 μm are giant LDs, observed commonly in liver and adipocytes, sometimes exceeding 10 μm in diameter. Two key pathways explaining LD growth exist: coalescence and Ostwald ripening [56]. Coalescence involves the direct fusion of membrane phospholipids and neutral lipids from two LDs into a single larger entity. Ostwald ripening is akin to a thermodynamic process and involves diffusion of the lipids in smaller particles into the cytoplasm and their absorption into larger ones. In adipocytes, Ostwald ripening is mediated by CIDEC/FSP27 and facilitated by accessory proteins Perilipin 1(PLIN1) and RAB8a [57]. Coalescence is accelerated under pyruvate carboxykinase deficiency conditions, in contrast to the slower Ostwald ripening process. The membrane phospholipids needed for Ostwald ripening and giant LD formation are synthesized by choline phosphotransferase on the LD surface. Choline phosphotransferase and phosphatidylcholine-sterol acyltransferase synthesize phosphatidylcholine and lysophosphatidylcholine directly on the LD surface. Ostwald ripening prevents simultaneous maturation of multiple droplets as equilibrium dictates that only one LDs matures, absorbing the lipids of smaller droplets [58]. As mentioned, seipin is crucial for LD formation. Studies have shown that acute seipin depletion causes small LDs to disappear and oversized ones to emerge. Seipin is proposed to act as a gatekeeper for Ostwald ripening and is crucial in LDs maturation [43]. Hepatic steatosis is characterized by giant LDs in hepatocytes. Phosphatidylcholine supplementation inhibits LD fusion, improving liver weight and serum aminotransferase levels in patients with NAFLD, and it supports VLDL secretion with lysophosphatidylcholine, reducing liver triglyceride accumulation and alleviating steatosis. Giant LD formation in adipose tissues is associated with macrophage-driven chronic inflammation. LD size varies significantly with cell status, ranging from 0.1 μm to 10 μm. Post-ER budding LDs that get stabilized in the cytoplasm are referred to as initial LDs (iLDs). iLDs measure approximately 0.3 μm to 0.8 μm [59], they can grow into various expanded LDs (eLDs) within minutes to hours after formation, depending on the status of the cell [60]. Under normal conditions, iLDs expand into eLDs via in situ synthesis. Cytoplasmic coating proteins ARF1/COP1 transform iLDs into 60–80 nm nanodroplets, increasing their membrane surface tension [54]. The interaction with these proteins fosters membrane bridge formation between LDs and the ER [61]. DGAT is transferred from the ER membrane to nanodroplets via membrane bridges, catalyzing triglyceride synthesis and promoting eLD formation.

### Degradation of LDs

Fatty acids are broken down either by lipases on the surface of LDs or within lysosomes by lysosomal acid lipase [62]. In mammalian adipose tissues, hormone-sensitive lipase (HSL) hydrolyzes triglycerides, whereas adipose triglyceride lipase (ATGL) initiates triglyceride hydrolysis [63]. Triacylglycerol is metabolized into diacylglycerol [63]. HSL, a lipase with broad substrate specificity, cleaves diacylglycerol into monoacylglycerol and fatty acids, thereby hydrolyzing triglycerides, monoacylglycerols, and cholesterol esters [64]. Monoglyceride lipase ultimately breaks monoacylglycerol down into glycerol and fatty acids [65]. Under nutrient deficiency conditions, adipocytes undergo lipolysis and lipophagy, regulated by adenosine monophosphate-activated protein kinase (AMPK) and mammalian target of rapamycin complex 1 (mTORC1). This pathway decomposes LDs, releasing free fatty acids to energize other tissues [66]. Chaperone-mediated autophagy (CMA) precedes and enhances both lipolysis and lipophagy [67]. Research indicates that RUBCN negatively regulates autophagy [68], while mTORC1 function decreases in adipocytes throughout senescence and caloric restriction [69]. Under certain conditions, autophagy upregulation in adipocytes may inhibit MTORC1 activity and affect the transcriptional regulation of RUBCN.

### LDs and autophagy

Autophagy, an evolutionarily conserved mechanism, involves eukaryotic double-membrane vesicles encapsulating intracellular contents for lysosomal degradation [70]. A significant link exists between lipid metabolism and cellular autophagy mechanisms. Lipid derivatives activate the mTOR signaling pathway, inhibiting autophagy initiation. Under nutrient-rich conditions, activation of the nutrient-sensitive kinase mTORC1 stimulates the biosynthesis of proteins, lipids, and nucleosides, inhibiting autophagy. Conversely, autophagy is promoted under nutrient scarcity [71]. On the cell membrane, different lipid molecules bind to specific effector proteins, influencing intracellular vesicle morphology and movement. Autophagy initiation relies on the synthesis of phosphatidylinositol 3-phosphate [72]. Additionally, some lipid molecules promote protein modifications, including the lipidation of the ATG8/LC3 protein family, facilitating autophagic vesicle expansion and autophagosome formation [73]. Moreover, the distribution of lipid molecules within the bilayer membrane, including the accumulation of phosphatidic acid, diacylglycerol, and ceramide, regulates autophagy by promoting autophagy-associated vesicle growth and fusion.

Lipophagy, the selective autophagy of lipid compounds, orchestrates lipid catabolism. Within cells, LDs are the typical storage sites for lipids. Triglycerides and cholesterol in LDs are metabolized in mitochondria via β-oxidation to produce ATP, ensuring a continuous supply of cellular energy. Lipophagy regulates fat metabolism via different mechanisms: (1) Suppression of autophagy results in elevated lipid levels in LDs. (2) During lipophagy, LC3 targets the membrane of LDs. Involvement of the SNARE complex, related to soluble N-acetylglutamate, may facilitate LDs’ association with autophagic vacuoles. (3) ATG15 is posited to act as a lipase [74].

Contrary to the widespread belief that starvation accelerates lipid catabolism, prolonged nutritional deficiency actually increases LD accumulation, a process not observed in mouse embryonic fibroblasts lacking the essential autophagy gene ATG5 [75]. LDs dynamically regulate lipid metabolism, capturing excess fatty acids to mitigate lipotoxicity [76]. Although the precise cause of LD accumulation during starvation remains unclear, data from Truc B Nguyen suggests that mTORC1-regulated autophagy participates in this process, potentially reducing mitochondrial lipotoxic damage [77].

The corpus luteum, a unique and transient structure, is vital for female reproductive function. BECN1 is a key autophagy regulator that promotes the storage of neutral lipids in corpus luteum cells. Its gene knockout in mouse ovarian granulosa cells significantly reduces LD accumulation in luteal cells post-ovulation [78]. In fruit flies, BECN1 assembles into conserved complexes with VPS15, UVRAG, and Bif-1, which are essential for the merging of autophagosomes, endosomes, and lysosomes. However, the hypothalamic-pituitary-gonadal (HPG) axis remains unaffected by BECN1 deficiency in luteal cells. In Drosophila larvae fat body tissues, *Atg1* gene overexpression inhibits cell growth by negatively regulating the Tor signaling pathway. Researchers have identified 18 RAB proteins that influence LD size. Specifically, RAB32 mutants exhibit smaller LDs and decreased autophagy activity in fat bodies than their normal counterparts. Since RAB32 is primarily localized on autophagosomes, not in LDs, the protein may regulate LD size by influencing autophagy [79].

## Lipid droplet-associated protein

LDs are associated with over 200 proteins of various forms and functions that collaboratively regulate the droplets’ formation, growth, stability, and metabolism [80]. These proteins can be broadly categorized as follows: structural associated with the LDs membrane (e.g., PLIN and LSD2) that protect LDs and regulate their function; lipid metabolism enzymes on the surfaces of the ER and LDs (e.g., DGAT1 and HSL) that synthesize and degrade neutral lipids; membrane transport proteins on the the surfaces of the ER and LDs (e.g., RAB18) that regulate interactions between LDs and other cellular structures; signaling proteins (e.g., MAPK) involved in inflammation and signal transduction; and degradation-related proteins (e.g., UBXD8) that degrade LD-associated proteins. Additionally, other proteins, such as ribosomal proteins, histones, and actin, are involved in protein translation, chromosome assembly under stress, and LD movement, respectively.

These proteins are essential for maintaining a normal lipid metabolic balance in LDs, and their dysfunctions are linked to metabolic disorders like obesity, diabetes, and cardiovascular conditions [3]. Table 1 lists LD-associated proteins, including RAB, PLIN, and CIDE emphasizing their critical functions in LDs formation and the regulation of their sizes and numbers.

**Table 1:** Partial lipid droplet-associated proteins

Protein Classification	Protein Name	Function
RAB	RAB1	Participates in the biosynthesis and transport of proteins regulated by the ER and Golgi apparatus.
	RAB5	Functions as a regulator between early nuclear bodies and lipid droplets
	RAB8	Cell shape regulator.
	RAB11	Participation in cytoplasmic division, cell migration, immunological synapse, control of the endosomal recycling of β-secretase.
	RAB18	Involved in GTP cycling, lipolysis, autophagy, and ER metabolic transport.
PLIN	PLIN1	Involved in lipolysis, bidirectional regulation of lipid metabolism, and interaction with small CAV1 to coordinate the transfer of exogenous fatty acids.
	PLIN2	Participation in hepatocyte autophagy, triglyceride metabolism, and enhancement of the activity of anti-inflammatory genes.
	PLIN3	Localized on the surface of LDs and mediating endosomal transport. Deficiency in this mechanism may stimulate the activity of beige fat cells.
	PLIN4	Formation of nascent micro LDs.
	PLIN5	Maintenance of the equilibrium between lipid synthesis and lipolysis, and regulation of fatty acid oxidation in oxidative tissues.
CIDE	CIDEA	To reduce lipid breakdown, specific E3 ubiquitin ligases might be targeted to AMPK-β, leading to its ubiquitin tagging and subsequent proteasome-mediated degradation.
	CIDEB	Growth and fusion of LDs in hepatocytes, it can stimulate cholesterol synthesis and may decrease insulin sensitivity.
	CIDEC	The three types: α, β, and γ, participate in cellular apoptosis, promote the formation of unilocular LDs, and inhibit lipolysis

### RAB Protein

In proteomics studies, RAB proteins, a class of small GTPases involved in intracellular solute trafficking, have been identified on LDs [20, 81, 82]. Despite minimal sequence homology, RAB proteins typically feature conserved GTP hydrolysis binding sites, including serine and threonine residues at the N-terminus that enable them to function as GTP switches. RAB proteins regulate membrane docking and vesicle movement, thereby modulating intracellular membrane trafficking [83]. The RAB proteins localized on LDs include RAB1-5, 7, 8, 10–14, 18, 19, 21, 22, 31, 34, 35, 39, and 40. [84]. RAB18 is consistently present throughout the LD lifetime, warranting special attention.

#### RAB18

RAB18 is among the conserved RAB proteins facilitating the interaction between mature LDs and the ER [85, 86]. Insulin stimulates RAB18’s recruitment to LD surfaces via phosphatidylinositol 3-kinase activation. Moreover, RAB18 overexpression enhances basal lipogenesis, whereas its knockdown impairs insulin’s lipogenic response, suggesting the protein promotes adipocyte fat accumulation [87]. Patients lacking RAB18 with Warburg-Micro syndrome exhibit severe symptoms including microcephaly, congenital cataracts, agenesis of the corpus callosum, profound intellectual disability, and hypogonadism [88].

RAB18 participates in the viral replication and morphogenesis of viruses, including BK polyomavirus, dengue virus, and hepatitis B virus [89, 90]. RAB18 may be involved in transporting fatty acid synthase and nonstructural protein 3, thereby enhancing dengue virus replication in A549 cells [89]. RAB18 expression levels positively correlate with hepatitis B virus presence in clinical liver cancer tissues. HCV’s core protein assembly onto LDs relies on RAB18, linking the protein to LD-ER interactions [91].

RAB18 participates in GTP recycling, lipolysis, autophagy, and ER metabolic transport. The mechanisms involving RAB18 in lipolysis regulation via autophagy upregulation and virus assembly require further investigation [92].

#### Other RAB proteins

RAB1 regulates protein biosynthesis and transport between the ER and Golgi apparatus, extending beyond vesicle transport to include nutrient sensing and cellular signaling [93]. RAB5 regulates interactions between early endosomes and LDs, requiring GTP for translocation from the cytosol to LD surfaces [94]. Like RAB18, RAB5 and RAB7 are involved in HCV assembly [95]. RAB7 is involved in endosome maturation and, when mutated, may result in Charcot-Marie-Tooth type 2B disease, though the specific mechanisms remain unclear [96]. RAB8 regulates cell shape, with its activation influencing actin-containing structures such as pseudopods, protrusions, and ruffles [97]. RAB8a facilitates LDs fusion; the SNARE protein, crucial for cell membrane fusion, is also present on LDs [98]. RAB11a is the most widely expressed gene of RAB11 [99] and is mechanisms including cytokinesis, cell migration, and immune synapse formation [100].

### Perilipins

Perilipins, named after the Greek ‘perilipin’, localize specifically on the LD’s surface when ectopically expressed in adipocytes and various other cell types [101]. The LIN family comprises five members (PLIN 1–5), with all except PLIN1 present in skeletal myocytes. The distinct roles of PLIN proteins in skeletal myocytes are unclear, with evidence suggesting their involvement in lipid storage, breakdown, and transport [102]. In 2017, Shepherd et al. showed that in type I muscle fibers, individuals who train regularly have a greater association of PLIN2, PLIN3, and PLIN5 with LDs. These individuals experience an increase in triglyceride content, which is associated with an enlargement and density of LDs. However, in sedentary individuals, the number of lipid droplets increases, but these droplets lack PLIN2, PLIN3, and PLIN5. The density of lipid droplets only increases following lipid injection. [103]. The perilipin family is primarily characterized by sequence homology in their N-terminal regions. The N-terminus region is referred to as the PAT domain, for ‘perilipin, adipophilin, TIP-47’, corresponding to PLIN1, PLIN2, and PLIN3, respectively [104]. Following the PAT region, all PLINs have variable-length 11-aa repeat regions along their sequence, which, although anticipated to be intrinsically disordered in solution, form amphipathic helices on lipid surfaces [105, 106].

#### PLIN1

PLIN1 is primarily mainly found in adipocytes within fat as well as breast tissues, with reduced expression noted in the steroidogenic and hepatic cells of patients diagnosed with non-alcoholic steatohepatitis (NASH) [107]. Steroidogenic cells are specialized cells within the body tasked with the biosynthesis and release of steroid hormones. Dynamic LD changes in steroid cells are associated with lipid metabolism diseases. For example, the trafficking of cholesterol-rich LDs in steroidogenic cells serves as the primary source of cholesterol for steroidogenesis [108]. PLIN1 thoroughly investigated regarding its function in lipid metabolism. This is a highly coordinated process involving the esterification of lipids and their breakdown into fatty acids [109]. ATGL, referred to as Patatin-like phospholipase domain containing 2 (PNPLA2), catalyzes the lipolysis cascade in adipocytes.

ATGL is modulated through interactions with multiple proteins, mainly through comparative gene identification 58 (CGI58). Under resting conditions, PLIN1 binds to CGI58 on the LD surface, making ATGL inaccessible. PKA phosphorylates PLIN1 upon stimulation of lipolysis through the activation of the cyclic AMP-dependent protein kinase A (PKA) pathway [110], CGI58 [102], and HSL. Phosphorylation of PLIN1 disrupts its interaction with CGI58, enabling CGI58 to translocate ATGL to the LD membrane [111, 112].

HSL recruitment to the LD surface enhances lipolysis [113]. Through its direct interaction with HSL and CGI58 and its indirect promotion of ATGL activity, PLIN1 acts as a master regulator of lipolysis. Ablation of PLIN1 increases basal lipolysis rates and reduces β-adrenergic stimulation effects on lipolysis, aligning with its regulatory role [114, 115]. Increased thermogenesis and adipose tissue browning in PLIN1 knockout mice are attributed to high basal lipolysis rates [115, 116]. PLIN1 facilitates autophagy protein recognition and the breakdown of LDs during lipophagy, offering an additional mechanism for LD turnover coordination [117]. By promoting the incorporation of fatty acids, PLIN1 may enhance LD synthesis and growth, contrary to its role in lipolysis inhibition. The interaction of PLIN1 and caveolin 1 (CAV1) in caveolae—specialized plasma membrane areas—facilitates the incorporation of external fatty acids into triglycerides in LDs [118, 119]. PLIN1’s interaction with CIDEC, or fatty acid-binding protein 27 (FSP27), promotes LD fusion, facilitating white adipocyte-specific monocular LD formation [120]. A result of their elevated basal lipolysis rate, PLIN1-deficient mice exhibit resistance to diet-induced obesity [115].

PLIN1 regulates lipid metabolism bidirectionally [121]. The removal of PLIN1 decreases lipid synthesis gene expression and increases oxidative gene expression. PLIN1 gene knockout mice show obesity resistance, potentially due to inflammation of tissue inflammation and insulin resistance, driven by increased recruitment of inflammatory macrophages [122].

Interestingly, PLIN1 overexpression causes adipose tissues to shrink and imparts resistance to diet-induced obesity [123, 124]. Despite the similar anti-obesity effects in knockout and overexpression models, PLIN1 adipose overexpression enhances glucose tolerance and insulin sensitivity, but its deficiency causes the opposite effects in mice [123]. These studies indicate PLIN1’s essential function in regulating lipid homeostasis and insulin sensitivity in adipose tissue. However, the exact mechanisms linking PLIN1 to lipid metabolism and insulin signaling pathways require further investigation.

#### PLIN 2

PLIN2, also referred to as differentiation-related protein (ADRP), belongs to the PAT family, which coats LDs. Despite its inability to interact with lipolysis-related proteins, PLIN2 can decrease the production of LDs or triglycerides [125, 126]. Autophagy seems to be an alternative route for PLIN2 and triglyceride degradation in hepatocytes, facilitating LD breakdown [67].

On a high-carbohydrate diet, PLIN2 knockout mice show reduced energy intake and lower blood glucose levels than controls. This condition also leads to brown adipose tissue whitening, contributing to obesity resistance in mice [127]. PLIN2 has been shown to affect the expression of the intestinal microflora; and, mice lacking PLIN2 exhibit decreased intestinal fat absorption and fewer LDs [128]. This suggests that PLIN 2 can affect energy metabolism.

PLIN2 expression increases in response to inflammatory stimuli like lipopolysaccharide from Gram-negative bacilli, potentially via P38 mitogen-activated protein kinase (P38 MAPK) activation [129]. Within aged microglia, large LDs correlate with increased PLIN2 expression [130]. PLIN2’s degradation pathway markedly lowers the inflammation associated with a high-fat diet, preventing atherosclerosis formation. PLIN2 binds various lipids, notably cholesterol and arachidonic acid, with high affinity. PLIN2 deficiency may increase lipolysis and promote fatty acid mobilization via through upregulated peroxisome proliferator-activated receptor gamma (PPAR) signaling pathways [131]. PPAR signaling pathways significantly affect the metabolism and boost the activity of anti-inflammatory genes [132], a fact that highlights the benefits obtained after PLIN2 deletion.

RAB 18 interacts with PLIN2 in the formation of a complex with ACSL3, crucial for LD metabolism in myoblasts [133].

#### PLIN 3

Some perilipins localize predominantly on the surface of LDs, where they probably exert their functions. The initial studies on PLIN3 were controversial; however, further research confirmed its role mediating endosomal transport [101]. PLIN3 binds to diacylglycerol-containing membranes through conserved residues in its PAT domain [104]. PLIN3 binds to liposomes with low diacylglycerol concentrations, as mentioned in the LD formation section. PLIN3 is crucial for ER formation and accumulates in the subdomain involved in LD formation [134]. When PLIN 3 is knocked down, the LD formation is inhibited [135], preventing diet-induced hepatic steatosis and reducing VLDL secretion [136]. In cultured adipocytes, PLIN2 replaces PLIN3 as LDs mature. In most non-adipocyte cells, medium and small LDs are abundant in both PLIN2 and PLIN3 molecules [137].

Cell culture experiments indicate that PLIN3 knockout in mouse AML-12 cells leads to formation of small LDs covered by PLIN2 without affecting triglyceride storage. Conversely, high expression of PLIN2 and PLIN3 decreases triacylglycerol storage and enlarges LDs. These studies demonstrate PLIN’s role in minimizing the cytosolic surface area and PLIN3’s regulation of phospholipid metabolism [137]. PLIN3 competes with cytidine triphosphate phosphatidyl transferase-α (CCTα), also known as choline phosphatidyl transferase 1 A (phosphate cytidylyltransferase 1 A). PLIN3 binds to nuclear LDs, where CCTα, a critical enzyme involved in the biosynthesis of phosphatidylcholine, can bind to enhance the synthesis of phosphatidylcholine. Thus, PLIN3 may reduce phosphatidylcholine synthesis by competing with CCTα on nuclear LDs. Under fasting conditions, PLIN3 undergoes phosphorylation by AMPK and choline kinase α2, leading to its degradation via CMA [138]. PLIN3 deficiency activates PPARα, stimulating thermogenic beige adipocytes [139].

PLIN3 facilitates LD formation; its degradation accelerates droplet breakdown, restricts nuclear thermogenic genes in lipid metabolism, and prevents the formation of nonfunctional beige fat cells.

#### PLIN 4

PLIN4’s 11-mer repeat at the extended amino terminus shows significant sequence diversity and minimal homology with other family members. The repeated sequences likely form amphipathic α-helices, positioning nascent proteins on LDs [15].

Perilipin 4 expression is most abundant in white adipose tissue, while reduced levels are observed in the cardiac and striated muscle [140]. PLIN4, particularly in generating small new LDs in cultured adipocytes, remains unclear [141]. Moreover, PLIN4 contributes to LDs formation in white adipose tissue, heart, and skeletal muscle, yet is not involved in brown adipose tissue. In the mouse genome, the *Plin4* gene, positioned directly downstream of *Plin5*, shows unique yet overlapping expression patterns with it. Studies indicate that deleting the *Plin4* gene in mice reduces triglyceride levels in the heart. Inactivating *Plin4* reduces *Plin5* expression and cardiac lipid accumulation in mice [140], without affecting fat content. *Plin4* knockout did not significantly alter white adipose tissue function, possibly due to reduced PLIN5 expression in this tissue. A study on Parkinson’s disease (PD) showed that PLIN4 downregulation decreased LDs storage, thereby enhancing survival in the SH-SY5Y human neuroblastoma cells and indicating a dysfunctional PLIN4/mitophagy axis in PD’s pathogenesis. This suggests PLIN4-LD could be a potential PD biomarker, although further research on PLIN4’s functions is needed.

#### PLIN 5

PLIN5 balances lipogenesis and lipolysis processes, and it regulates fatty acid oxidation in oxidative tissues. PLIN5 recruits mitochondria to LDs, regulating triglyceride storage and fatty acid release for mitochondrial oxidation, thereby maintaining LD homeostasis. PLIN5, like other apolipoproteins, binds to LD surfaces, protecting them from degradation. PPARα transcriptionally regulates PLIN5, which is discovered in the myocardium, red muscle fibers, and liver tissue, all of which are involved in fatty acid oxidation [142]. *Plin5* and *Plin2* genes share similar transcriptional regulators and are jointly regulated in the cardiac and hepatic tissues in response to physiological changes, such as fasting and refeeding [143].

PLIN5 potentially functions bidirectionally in the dynamic equilibrium within lipid metabolism. Under alkaline conditions, PLIN5 binds to CGI58, inhibiting its interaction with ATGL and preventing lipolysis. Under stressful stimuli like cold exposure, fasting, and exercise, PKA phosphorylates PLIN5, exposing binding sites for CGI58 and ATGL, thereby promoting their interaction and activating ATGL to enhance lipolysis [144].

Autophagy represents a highly conserved cellular self-protection mechanism across evolution. Meanwhile, mitophagy maintains intracellular homeostasis by clearing labeled mitochondria and their components. Selective autophagy balances lipid production through the regulation of lipid biosynthesis and breakdown [67, 145]. Monounsaturated fatty acids are the first identified endogenous SIRT1 allosteric regulators. They bind to PLIN5, activating the *SIRT1* gene, which in turn enhances PGC-1α/PPARα signaling, promoting mitophagy and inhibiting inflammation [146]. Experiments indicate that PLIN5 inhibits lipolysis, mitigates oxidative stress, and diminishes myocardial ischemia/reperfusion injury via enhanced phosphorylation of PI3K/Akt [147].

### CIDE

The full name of CIDE is The cell death inducing DFF45 like effect. The protein family regulates cell apoptosis as well as fat metabolism. It was initially identified due to its homology with the N-terminal domain of DNA fragmentation factor 40/45 (DFF40/45) [148]. Caspase-independent cell death (CICD) was soon found to be induced by CIDE [149]. Since its 1997 discovery, research on the CIDE protein family has surged in the fields of cell biology and metabolism. These proteins are crucial for cellular metabolic homeostasis and disease development. There are three main types of CIDE proteins: CIDEA, CIDEB, and CIDE3 (also known as CIDEC or FSP27). All CIDE members localize to LDs, promoting their growth and the formation of large LDs to enhance cellular lipid storage efficiency [150].

#### CIDEA

CIDEA expression is predominantly found in rodent brown fat. CIDEA converts excess energy to heat via adaptive thermogenesis, thereby regulating lipolysis and modulating lipid storage within brown adipose tissue depots [151]. In humans, CIDEA is present in both brown and white adipose tissues, storing excess energy as triacylglycerol, a fact that differs in rodents [152]. CIDEA regulates lipolysis in human adipocytes [153]. CIDEA as well as CIDEC are present in fatty livers with enlarged LDs [154]. The promoter activity of CIDEA is promoted by the activation of peroxisome proliferator activated receptor-γ co activator factor-1 alpha (PGC-1α), estrogen related receptor-α, and nuclear respiratory factor-1 [155]. Corepressor RIP140 inhibits PGC-1α, affecting CIDEA’s transcriptional activity [156]. CIDEA increases adaptive thermogenesis, potentially by binding to mitochondrial uncoupling protein 1. CIDEA co-localizes with AMPK and specifically interacts with its β subunit to form a complex with an unclear function. CIDEA might attract specific E3 ubiquitin ligase to AMPK-β, enhancing its ubiquitination and subsequent proteasomal degradation, thereby decreasing fatty acid oxidation [157].

CIDEA enlarges LDs and reduces lipolysis. Mice lacking CIDEA exhibit a lean phenotype with increased metabolic rates and higher body temperature. Studies indicate that hepatic CIDEA silencing in ob/ob mice livers significantly reduces lipid accumulation, resulting in smaller LDs compared to CIDEA overexpressing samples. Knocking down CIDEA improves fatty livers [158]. In a study of patients with gastric bypass surgery, CIDEA and CIDEC levels correlated strongly with body mass index (BMI), with reduced CIDEC expression post-surgery [159]. Sebaceous glands, which are cutaneous appendages secreting sebum into hair follicles, lubricate hair and maintain skin homeostasis [160]. High CIDEA expression in lipid-rich sebaceous gland cells is linked to hair dryness and loss in aged mice with CIDEA deficiency. Similarly, high CIDEA expression in human sebaceous glands positively correlates with sebum secretion [161]. During lactation, the mammary gland, a lipid-rich organ, secretes vital lipids for the offspring; CIDEA deficiency can lead to premature pup mortality due to insufficient lipids [162].

#### CIDEB

Cell death-inducing DFFA-like effector B (CIDEB) localizes to the LDs and ER, enhancing the growth and fusion of hepatocyte LDs [163, 164]. In addition to the expression in the liver, CIDEB is also abundantly expressed in the mouse small intestine, liver, and kidney [165]. CIDEB facilitates lipid storage in the liver by promoting LD fusions and growth. CIDEB knockout in mice increases energy consumption and reduces body fat, serum triglycerides, and free fatty acids contents [166]. CIDEB is also involved in the cholesterol metabolism pathway. On a normal diet, cholesterol levels in CIDEB-deficient mice are reduced by 60% compared to wild-type mice. Under a high-fat diet, cholesterol levels in CIDEB-deficient mice are 66% lower than in wild-type mice [167]. CIDEB significantly regulates cholesterol synthesis and absorption.

CIDEB-deficient mice exhibit increased insulin sensitivity. Under normal diets, these mice exhibit reduced glucose levels and 50% lower insulin levels than normal mice. Under a high-fat diet, the reduction in insulin levels reaches 80% [166]. Hepatic lipid homeostasis is maintained by CIDEB by promoting the loading and export of the SREBP/SCAP to ER exit sites. CIDEB’s interaction with Sect. 12 concentrates SCAP/SREBP at ER exit sites, thereby facilitating COPII vesicle formation [168]. In summary, CIDEB has multifunctional roles regulating hepatic lipid secretion, storage, and synthesis.

The pathogenesis of ulcerative colitis (UC) is contributed to by lipid metabolism. Expression of CIDEB diminishes triglyceride accumulation and alleviates oxidative stress in Caco-2 cells exposed to oleic acid, offering potential benefits for patients with UC. This suggests a protective role against UC [169]. The assembly of the Hepatitis C Virus (HCV) is related to CIDEB, which acts as a crucial cofactor for the virus to penetrate hepatocytes. CIDEB may facilitate membrane fusion during HCV infection [170] and influence HCV’s binding to apolipoprotein E (APOE). Moreover, dengue virus infection also requires CIDEB [171].

#### CIDEC

CIDEC, also referred as FSP27, is a member of the cell death-inducing DFFA-like effector family. Initially identified in 3T3-L1 adipocytes as proteins associated with LDs, FSP27 encodes for a cell death-inducing effector that significantly contributes to apoptosis [172]. In mice, both brown and white adipose tissues exhibit a high level of CIDEC expression, whereas in humans, its expression is restricted to the white adipose tissue [173]. CIDEC forms homodimers at LD contact sites that mediate lipid transfer from smaller to larger droplets, propelled by the high internal pressure of the smaller droplets [174].

Like CIDEB, CIDEC enhances insulin sensitivity. A nonsense mutation, E186X, in CIDEC, has been associated with lipodystrophy, dyslipidemia, and insulin-resistant type 2 diabetes mellitus (T2DM) [175]. CIDEC upregulates the ATGL transcription, whereas its overexpression exerts an opposing effect in human adipocytes [176]. By interacting with CGI58, CIDEC modulates ATGL’s catalytic activity, restricting free fatty acid release [175]. In human, the interaction between CIDEC and ATGL regulates lipid degradation, triglyceride accumulation, and insulin signaling pathways [177].

In CIDEC-deficient mice, white adipocytes show reduced triglyceride levels and small LDs. CIDEC promotes single-chamber LD formation, limiting lipolysis and enhancing energy storage in white adipose tissues [178]. Lipolysis in mouse adipocytes correlates with CIDEC downregulation [179].

PLIN1 is associated with CIDEC, promoting CIDE-mediated LD fusions. This interaction depends on the acidic residues of both PLIN1 and CIDEC. The cyclic arrangement of six lysine residues within the N-terminal region significantly promotes LD growth [120].

FSP27 has a subtype, FSP27β, which contains an additional 10 amino acids at the N-terminus relative to FSP27α [180]. FSP27α is uniquely expressed in white adipose tissue, and FSP27β, on the other hand, is uniquely expressed in both the liver and brown adipose tissue. FSP27β inhibits CIDEA homodimerization in brown adipose tissues, a key step to forming small multicompartmental LDs, regulated by liver-enriched CREBH [180, 181]. PPARγ induces FSP27α expression through a specific cis-element [181]. PPARγ operates via a common PPAR response element (PPRE). FSP27 knockout mice show reduced oxygen consumption in isolated brown adipose tissues relative to that observed in wild type mice [178]. Within white adipose tissues, the formation of numerous small LDs by FSP27β suggests increased oxygen consumption. Thus, FSP27β’s role in LD formation requires further studies on adipocyte metabolism [181].

## Lipid droplet-related proteins and diseases

**Fig. 2:**
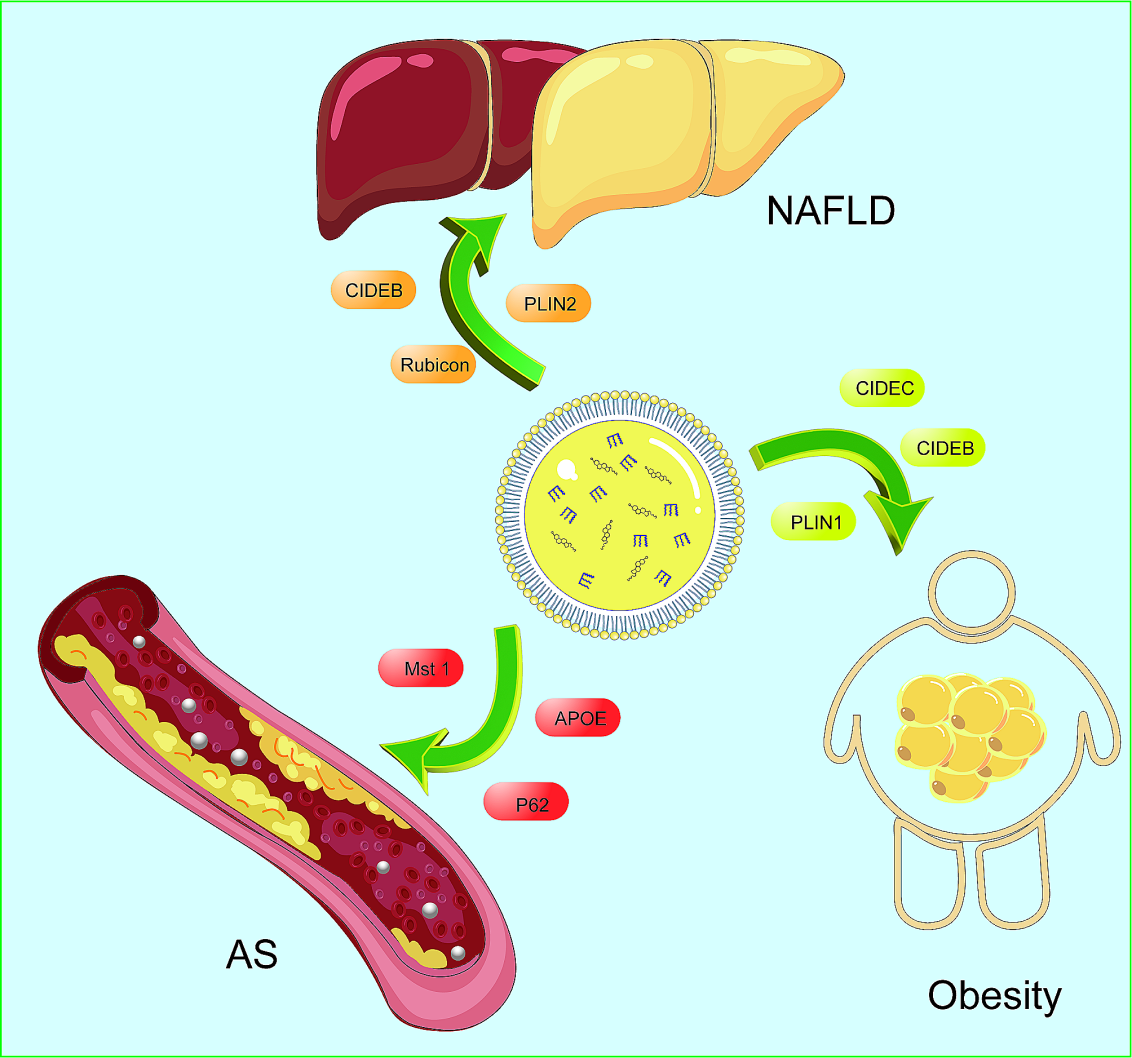
Associations of LDs with diseases. 1. LDs and NAFLD: The highly expressed PLIN1, which can inhibit lipolysis and autophagy, and the upregulated PLIN2, are key players in the regulation of NAFLD. Additionally, genes such as RUBCN and CIDEB modulate the disease. 2. LDs and obesity: CIDEB and CIDEC can promote insulin sensitivity, whereas PLIN1 and CIDEC regulate lipolytic enzymes such as HSL, promoting the progression of obesity. 3. LDs and atherosclerosis: LD-associated proteins, including those in the PLINP (perilipin) family and apolipoprotein APOE, participate in the development of atherosclerosis. P62 regulates autophagy, influencing the progression of the disease. Cholesterol acyl transferase 1 (ACAT 1) and APOE are both regulators of cellular cholesterol homeostasis and have a crucial role in the formation of foam cells

### Obesity

Triglyceride synthesis, storage, and breakdown are crucial steps in adipocytes for maintaining the lipid balance in living organisms. LDs participate in lipid metabolism and have a role in obesity via metabolic disorders.

Obesity is marked by the accumulation of excessive fat within adipocytes, muscle cells, and organ cells, reflecting increased LD contents. The BMI is a common measure of obesity. By dividing a person’s weight in kilograms by the square of their height in meters, it is calculated.

Studies on patients with obesity and T2DM have shown a positive correlation between PLIN1, CIDEC, and BMI in abdominal subcutaneous (SAT) and visceral adipose tissues [182]. HSL mediates triacylglycerol hydrolysis, regulated by PLIN. PLIN-knockout mice show a 6.2% reduction in white adipose tissue and increased basal lipolysis, leading to enhanced leptin production and lipolysis [114, 115]. The interaction between CIDEC and PLIN1 may facilitate LD fusions, suggesting a potential mechanism [120]. Obesity has also been correlated with the onset of conditions like T2DM, NAFLD, cardiovascular disease, and malignancies. LD-related proteins, such as CIDEB and CIDEC, may influence insulin sensitivity (Fig. 2) [183, 184]. Spalding et al. found that the number of adipocytes is similar between individuals with leanness and obesity; and that fat content differences are based on the size of adipocytes [185]. This correlates with LD size and has metabolic implications that require further investigation to unveil new targets for obesity treatment.

Fat synthesis and breakdown are closely linked to the body’s lipid balance, with lipophagy activity increasing as fat content rises [186]. Insulin resistance is more pronounced in individuals with obesity as a compensatory mechanism to mitigate the lipotoxic effects of excess adiposity [187]. Lipophagy activation varies individually: in obese patients, insulin feedback via insulin receptor substrate-1 (IRS-1) is weakened, mitochondria are damaged, and autophagy is significantly upregulated. The autophagic degradation of mitochondria and lipofuscin granules, coupled with ATP production’s reliance on autophagy, weakens insulin activation of mTORC1, enhances autophagy, and reduces feedback on IRS-1 [188]. Another critical aspect of obesity is inflammation. In macrophages, SREBP-1a and LXRα are highly expressed, they regulate cytokine release. Enlarged LDs may increase chronic inflammation due to cell remodeling and SREBP-1a activation [188, 189]. Interventions that reduce LD formation and size can alleviate obesity-induced inflammation. Obesity also confers psychological problems on those affected by it. A high-fat diet-induced obesity may suppress autophagy through the reduction of AMPK phosphorylation and the enhancement of mTOR phosphorylation, leading to depressive and anxiety-like behaviors in mice. Therefore, targeting lipid metabolism or boosting autophagy through the AMPK/mTOR pathway could serve as potential therapeutic strategies for managing obesity-related depression. [190].

In obese individuals, the number of adipocytes remains relatively stable. However, adipocyte numbers proliferate during childhood and early adulthood. Thus, a homeostatic control over the formation and elimination of adipocytes should be beneficial. Whether this homeostasis can also be applied at the LD level remains unknown. This could be crucial for the clinical relevance of the adipose tissue expansion hypothesis [191].

### Diabetes

Insulin resistance correlates with the accumulation of neutral lipids in non-adipose tissues [192]. LDs with their bound proteins are crucial for lipid metabolism, cellular energy balance, protein transport, and stress responses. In T2DM, the abnormal accumulation of LDs in non-adipose tissues leads to insulin resistance [193]. Fatty acids within LDs interfere with insulin signaling pathways through various mechanisms, decreasing cellular sensitivity to insulin. As mentioned in the obesity section, proteins like PLIN1 and CIDEC affect leptin production and insulin sensitivity. Muscle fiber type differences also affect LD morphology, localization, and insulin sensitivity [194]. The primary mechanism connecting obesity to metabolic complications involves the adipose tissue’s ability to expand and store excess nutrients. Studies on athletes and patients with T2DM indicate differences in lipid storage. In patients with T2DM, lipids predominantly accumulate in a few large droplets within the subsarcolemmal region of type II fibers, whereas in athletes, lipids are stored in numerous small droplets in the myofibrillar (MF) area of type I fibers. PLIN2 is negatively correlated with insulin sensitivity, whereas ATGL and PLIN5 are positively correlated with it. In athletes, changes in these proteins reflect increases in insulin sensitivity: PLIN2 decreases, while ATGL and PLIN5 increase [184].

The roles of LD proteins in insulin sensitivity remain unclear. Several studies indicate that soluble N-ethylmaleimide-sensitive factor attachment protein receptors (SNARE) significantly affect LDs’ impact on insulin sensitivity. Among them, the prominent ones are the 23 kDa SNARE protein and soluble NSF binding protein (SNAP23), which participate in the process of lipid droplet fusion. Oleic acid treatment reduced insulin sensitivity in cardiomyocytes, but the sensitivity was fully restored by transfection with SNAP23 [195]. By inducing the translocation of glucose transporter 4 (GLUT4) from intracellular vesicles to the plasma membrane, insulin facilitates glucose uptake in skeletal muscle and adipose tissue, a process significantly associated with SNAP23 [196]. Lipid accumulation in cells redistributes SNAP23 redistribute from the extracellular space to the intracellular space, resulting in insulin resistance [195].

The “athlete’s paradox” highlights the fact which LD size correlates positively with insulin resistance in patients with T2DM, but it correlates negatively with insulin resistance in trained athletes. Although abnormal LD accumulation decreases insulin sensitivity, LD formation can sequester free fatty acids, mitigating their impact and protecting the body. Variations in the levels of LDs proteins contribute to this paradox. The mechanisms by which LDs and their associated proteins affect diabetes mellitus are not fully elucidated. LD-associated proteins could be promising targets for future diabetes mellitus treatments and drug development.

### Atherosclerosis

Atherosclerotic plaque buildup in arteries can lead to significant cardiovascular diseases. Foam cells in the arterial wall remove modified LDL particles, causing predominantly LDL-derived cholesterol esters to accumulate in cytoplasmic LDs [197]. Cholesterol acyltransferase 1 (ACAT1) and APOE regulate the cellular cholesterol balance and are crucial in foam cell development [198].

Within the PAT protein family, adipogenic differentiation-related protein (ADFP) regulates the formation of foam cells and helps the development of atherosclerosis. Interactions between LDs and the ER lead to foam cell formation in atherosclerosis. LDs, mitochondria, and lysosomes influence cardiomyocyte remodeling via reactive oxygen species production and PI3K/AKT pathway regulation [199]. Further studies indicate that apolipoprotein E-deficient (APOE (-/-)) mice exhibit PLIN2 gene inactivation, reducing LD numbers in foam cells within atherosclerotic lesions, potentially protecting against atherosclerosis [200].

Atherosclerosis can be alleviated through cholesterol removal or inflammation resolution. Autophagy regulates the clearance of LDs and the outward transport of cholesterol from macrophage foam cells, facilitating reverse cholesterol transport and protecting against atherosclerosis [201]. p62 levels are elevated in atherosclerotic plaques. Mst1 deficiency markedly increases Beclin 1 and LC3 II levels, but decreases p62 in aortic atherosclerosis [202]. Autophagy activity is higher in the early stages of atherosclerosis and diminishes during later stages. Dysfunctional autophagy in atherosclerosis contributes to the disease’s progression by overactivating inflammasomes [203].

Multiple LD-associated proteins in macrophages contribute to foam cell development in atherosclerosis. Targeting the homeostasis of LDs and their associated proteins may represent new strategies against atherosclerosis (Fig. 2). Autophagy promotes macrophage lipid phagocytosis, serving as a crucial protective mechanism against atherosclerosis. Future research should aim to selectively enhance and monitor extracellular and intracellular lipid phagocytosis to refine this therapeutic approach. Clarifying the macrophages’ role in lipid processing and the efficacy of activating lipid phagocytosis could uncover new atherosclerosis treatments.

### Non-alcoholic fatty liver disease

NAFLD, a chronic disease linked to metabolic dysfunction, has been suggested by experts to be more aptly named Metabolic Associated Fatty Liver Disease ‘MAFLD’(Fig. 2) [204]. What causes NAFLD has developed from the single-hit hypothesis to the multiple-hit hypothesis. The latter suggests that intrahepatic fat accumulation, driven by factors such as physical inactivity, high-fat diet, obesity, and insulin resistance, represents the initial hit, predisposing the liver to further damage by the ‘second hit’. For instance, obesity in OB/OB mice often results in inflammation and fibrosis [205]. The contemporary multiple-hit theory includes insulin resistance, nutritional factors, hormones from adipose tissue, intestinal microbiota, and genetics as concurrent contributors to NAFLD development [206].

LDs accumulation in hepatocytes characterizes NAFLD. These droplets safeguard cells against excess free fatty acids by sequestering non-esterified free fatty acids as inert triacylglycerols, reducing their toxicity [207]. Studies indicate elevated expression of PLIN 1 in NAFLD patients, which inhibit lipolysis and autophagy [107]. Similarly, PLIN 2 is upregulated, correlating with increased ceramide accumulation in the liver [208]. A prominent feature of NAFLD is liver cell edema, where affected cells exhibit ER expansion and cytoskeletal damage [209].

Imbalances between class I and II proteins in the LD proteome can increase the number and accumulation of LDs. These imbalances may also facilitate LD fusions with each other or with organelles like the ER and mitochondria. These processes may result in heightened production of reactive oxygen species, exacerbate fat degradation in the liver, and potentially advance the progression of NAFLD to NASH [210]. Studies indicate that lipid phagocytosis is frequent in NAFLD. For instance, the protein RUBCN upregulates another protein that inhibits late-stage autophagy, and patients with NAFLD exhibit increased RUBCN levels [211]. Carbamazepine and rapamycin have been shown to enhance liver function and promote macro-autophagy in cells, contributing to NAFLD mitigation [212]. CIDEB promotes the growth and fusion of LDs, and SREBP is a key controller of lipid homeostasis. The absence of CIDEB results in reduced SREBP/SCAP loading decreased SREBP activation, and mitigation of diet-induced hepatic steatosis [168].

### Lipodystrophy syndromes

Lipodystrophy syndromes are disorders involving abnormal fat distribution and metabolic dysregulation. These syndromes are characterized by either excessive accumulation or deficiency of adipose tissue, often frequently presenting with metabolic abnormalities including insulin resistance, diabetes, hyperlipidemia, and hepatic steatosis [213]. Lipodystrophy syndromes are classified into generalized and partial types. Patients with generalized lipodystrophy display a marked decrease or complete lack of adipose tissue across the entire body, as seen in Berardinelli-Seip congenital generalized lipodystrophy (CGL) [214]. In contrast, patients with familial partial lipodystrophy (FPL) show uneven fat distribution, with some areas lacking adipose tissue and others possibly accumulating fat, as seen in Dunnigan-type FPL [215].

CGL typically presents at birth with a significant decrease in adipose tissue and an almost complete lack of white adipose tissue. Current reports subdivide CGL into four subtypes. Mutations in AGPAT2 cause type 1 CGL, while mutations in the BSCL2 gene, which encodes seipin, lead to type 2 CGL. These two subtypes are the most prevalent [216]. Type 3 CGL arises from homozygous nonsense mutations in the *caveolin-1* gene [217], whereas mutations in the polymerase I and transcript release factor gene cause type 4 CGL [218, 219]. Individuals with FPL exhibit varying degrees of subcutaneous fat loss, typically beginning at puberty or later, in the limbs and trunk. FPLD is categorized into eight types: the molecular basis of FPLD1 is unknown; FPLD2 is associated with missense mutations in *LMNA* [220]; FPLD3 is linked to heterozygous mutations in *PPARG* [221, 222]; FPLD4 is caused by heterozygous mutations in *PLIN1* [222]; FPLD5 is associated with homozygous nonsense mutations in *CIDEC* [223]; FPLD6 is related to homozygous mutations in *LIPE* [224]; FPLD7 is linked to heterozygous mutations in *ADRA2A* [225]; and the AKT-linked lipodystrophy subtype is associated with heterozygous mutations in *AKT2* [226].

LD-associated proteins are critical for adipose tissue function and LD formation, and their dysfunction is often linked to lipodystrophy syndromes. As mentioned, proteins such as AGPAT2, seipin, PPARG, PLIN1, and CIDEC are crucial for LD formation, growth, fusion, and metabolism. Understanding these proteins’ functions and mechanisms in lipodystrophy is crucial for uncovering these diseases’ pathogeneses and developing therapeutic strategies. Research advances have elucidated these proteins’ specific mechanisms in LD formation, providing new perspectives and potential therapeutic targets for lipodystrophy syndromes.

## Conclusions

LDs are considered functionally conserved organelles that have gained renewed interest in recent studies. Recent discoveries elucidating their complex formation, developmental processes, and protein interactions underscore the dynamic nature of LDs, highlighting their integral roles in numerous cellular functions. Comprehensive investigations into both traditional and specialized LDs (such as nuclear and peritubular variants) have uncovered the importance of associated proteins in multiple physiological and pathological processes. Our synthesis of the literature delineates the biosynthesis and metabolism of LDs, with a special focus on pivotal proteins such as RAB, PLIN, and CIDE, elucidating their structural and functional roles in detail. We described the intricate interactions among LDs, various organelles, and proteins and delved into their interconnected roles and mechanisms concerning obesity, autophagy, atherosclerosis, and NAFLD. In-depth research on the impact of LDs on metabolic diseases, using bioinformatics and molecular biology techniques should help to elucidate the functions of LDs and their associated proteins, thereby guiding the development of future pharmacotherapies and surgical approaches.

## Data Availability

No datasets were generated or analysed during the current study.
